# Models and analyses to understand threats to polio eradication

**DOI:** 10.1186/s12916-017-0991-5

**Published:** 2017-12-22

**Authors:** James S. Koopman

**Affiliations:** Deparment of Epidemiology, 1415 E. Washington Heights, Ann Arbor, MI 48109 USA

**Keywords:** Polio eradication, Polio transmission models, Dynamic system analysis, Complex system inference, Inference robustness assessment, POMP

## Abstract

To achieve complete polio eradication, the live oral poliovirus vaccine (OPV) currently used must be phased out after the end of wild poliovirus transmission. However, poorly understood threats may arise when OPV use is stopped. To counter these threats, better models than those currently available are needed. Two articles recently published in *BMC Medicine* address these issues. Mercer et al. (*BMC Med* 15:180, 2017) developed a statistical model analysis of polio case data and characteristics of cases occurring in several districts in Pakistan to inform resource allocation decisions. Nevertheless, despite having the potential to accelerate the elimination of polio cases, their analyses are unlikely to advance our understanding OPV cessation threats. McCarthy et al. (*BMC Med* 15:175, 2017) explored one such threat, namely the emergence and transmission of serotype 2 circulating vaccine derived poliovirus (cVDPV2) after OPV2 cessation, and found that the risk of persistent spread of cVDPV2 to new areas increases rapidly 1–5 years after OPV2 cessation. Thus, recently developed models and analysis methods have the potential to guide the required steps to surpass these threats. ‘Big data’ scientists could help with this; however, datasets covering all eradication efforts should be made readily available.

Please see related articles: https://bmcmedicine.biomedcentral.com/articles/10.1186/s12916-017-0937-y and https://bmcmedicine.biomedcentral.com/articles/10.1186/s12916-017-0941-2.

## Background

Polio eradication is the greatest global communitarian effort undertaken by humankind, thus failure to eradicate polio would be a global catastrophe. Nevertheless, eradication is complicated given the fact that success must be followed by complete global elimination of the oral poliovirus vaccine (OPV), without which, however, global eradication of polio is not possible. Elimination of the OPV is needed due to the threat of the poliovirus reacquiring wild poliovirus (WPV) characteristics and becoming circulating live vaccine-derived polioviruses (cVDPV), hindering full eradication of all pathogenic polioviruses. Thus, the Global Polio Eradication Initiative (GPEI) has been tasked with ensuring that all remaining OPV is destroyed after cessation of its use [1]. The three serotypes of WPV1–3 have varying characteristics, presenting different issues for polio eradication. WPV1 remains active and causing polio, whereas WPV2 and WPV3 were last detected in 1999 and 2012, respectively [2]. The elimination of WPV2 was relatively easy due to the high transmissibility of the OPV; however, this also led to a high rate of cVDPV2 emergence. WPV3 elimination has different dynamics because OPV3 is the least transmissible OPV and this could lead to prolonged silent circulation after the last polio case [2]. Global cessation of OPV2 occurred in April 2016. All trivalent OPV containing all three strains was destroyed and the standard OPV became bivalent OPV with only strains 1 and 3. Since then, cVDPV2 has been found in Nigeria, Pakistan, Syria, and the Congo [3].

## Modeling progress to achieve eradication

The combination of models and data can help eliminate the incidence of WPV1-caused cases and increase our understanding of how to reduce the likely threats arising from all three WPV strains upon cessation of their corresponding OPV. An article by Mercer et al. [4] in *BMC Medicine* addresses resource allocation to achieve the most efficient elimination of WPV1 cases in Pakistan. A further article by McCarthy et al. [5] addresses threat onset upon OPV2 cessation. These two papers advance methods within two different types of model analyses that can assist polio eradication. However, a third model analysis type (MAT) may be needed to insure eradication.

The MAT discussed here simplify a complex diversity of overlapping analytical methods into three categories. MAT1 uses non-mechanistic statistical models to analyze the associations between polio cases and predictor variables. This is the approach taken by Mercer et al. [4] MAT2 uses mechanistic models of how polioviruses are transmitted. These are constructed as mathematical models or computer simulations on the basis of established knowledge and/or assumptions. This is the approach taken by McCarthy et al. [5] Often, MAT2 parameters are adjusted to fit past patterns of polio cases without statistically estimating them. MAT3 fits mechanistic models to data in a more rigorous way, thus providing estimates of parameters. MAT3 can uncover the causal mechanisms generating observed patterns in data. Insights into causal mechanisms are essential to predict behavior in new situations that have never been experienced before. That is the case for polio eradication where we have never stopped OPV use before in situations where vaccination has decreased transmission for long periods without eliminating all cases. The technology used for MAT3 is new and developing rapidly.

Progress in all three model types could help ensure polio eradication. The first step toward eradication is achieving zero polio cases. This has not been easy in Pakistan. Mercer et al. [4] performed a MAT1 using data from WPV1 polio cases, together with past history and characteristics of local districts in Pakistan, to direct resources towards districts wherein they will most benefit eradication efforts. They made technical advances to MAT1 that improve descriptions of how surveillance variables track the risk of symptomatic infections. That, in turn, strengthens the GPEI strategy of learning how to refine eradication strategies by examining past experiences. Nevertheless, the data and the theory used by Mercer et al. cannot capture important issues that could arise when OPV use is stopped.

One such issue was examined by McCarthy et al. [3], who performed a MAT2 to model OPV2 cessation and the subsequent discovery of a cVDPV2 in such a population. The authors examined how local use of OPV2 to control the detected cVDPV2 could lead to the emergence of a new cVDPV2, which would subsequently spread to a population where OPV2 cessation remains in effect. Further, the authors showed that time since OPV2 cessation increases the risk of this occurring and examined how this risk is affected by migration rates, basic reproduction numbers, the transmissibility of OPV2 during its first stages of evolution into a cVDPV2, levels of immunity at the time of OPV2 cessation, and the use of inactivated polio vaccine. Additionally, the risk of cVDPV2 survival that could cascade out of control was seen to rise quickly with increasing time since OPV cessation, varying from 1 to 5 years as a function of the different factors assessed. Their study offers a useful advance in MAT2 methods by assessing the likelihood of an unfavorable outcome with built-in methods that simultaneously capture the effects of chance processes and the uncertainty of parameters. Their ‘separatrix’ graphs provide a comprehensible view of complex risk relationships. Given the ongoing cVDPV2 transmission in four locations now more than a year and half since OPV2 cessation, their result bring urgency to getting cVDPV2 under control.

The McCarthy et al. [3] model assumes there is no waning of immunity and that all transmission occurs in children under the age of 5 years with no immunity. These simplifying assumptions helped delineate mechanisms of cVDPV emergence and spread after OPV cessation. A next step to better understand such emergence and spread should realistically relax those simplifying assumptions. Doing so seems likely to further accelerate the spread of cVDPV2 after OPV2 cessation. Their assumptions of no waning and no transmission over age five are countered by adult cases in Tajikistan [6] and Xinjiang [7, 8]. These WPV1 outbreaks were caused by viruses geographically and genetically related to those in Pakistan. Additionally, confirmation of adequate prior OPV vaccination of adults in the 1988 Israeli WPV1 polio epidemic [9] specifically supports waning as a cause of those cases, as does the age pattern of dropping neutralizing antibodies in a population with high OPV coverage that eliminated WPV cases [10].

When these two assumptions are realistically relaxed, an important issue arises related to the generalizability of the inferences about high risk areas made by Mercer et al. [4]. The authors’ make no claims that their inferences about where efforts to prevent future cases will be most productive generalize to infections after the last case. Nevertheless, decision-makers might make such generalizations. This would be inappropriate because transmission can vary dramatically after detection of the last case depending upon how many transmissions arise from adults with waned immunity [11]. If waned immunity is sustaining silent circulation, areas with low rates of WPV or OPV transmission might have a higher chance of sustaining transmission than those identified by the type of analysis performed by Mercer et al. [4] since there would have been less frequent boosting of immunity in those areas.

Polio eradication is now too close to elimination of all paralytic cases for new fundamental science to elucidate how polio immunity wanes and how that affects WPV or OPV circulation. Therefore, MAT2 analytic approaches cannot resolve how waning will affect either the phenomenon of cVDPV persistence studied by McCarthy et al. [3] or WPV persistence in the absence of detected polio cases [11]. Instead, new approaches to linking models and data using MAT3 model analyses are needed.

## Using models to infer waning immunity effects on threats to polio eradication

Since the beginning of eradication efforts, the GPEI has gathered data obtained from acute flaccid paralysis surveillance, special immunization activities, sequencing of polio isolates, environmental surveillance, and various special surveys. New MAT3 methods are able to employ all such data in a single analysis to fit the parameters of transmission system models and thus generate the knowledge required to ensure an effective course to polio eradication. An early development in these models involves approximate Bayesian computation using sequential Monte Carlo fitting [12, 13]. More recent and more powerful methods use a sequential Monte Carlo approach, termed iterated particle filtering [14]. These and other approaches are all included in the POMP package for R [15], which this year saw a notable advance enabling the direct fitting of genetic sequence data to models [16]. Other advances include new approaches to inference robustness assessment and identifiability analyses [17]. New models of immune response to infection and waning of immunity published in the past year also make the use of these MAT3 methods more informative [18].

The task of informing eradication endgame decisions must be completed by research from various groups and a combination of unique approaches to modifying previously developed models, fitting those models to available data in ways that generate new knowledge, and performing identifiability analyses that indicate what new data will be most informative for good decision-making. Openness will be a key to applying MAT3 analyses to polio eradication. The particular history of GPEI endeavors in the context of WHO bureaucracy has understandably not promoted openness. However, this critical juncture calls for change. We need to understand what is impeding access to polio eradication program data and take action to improve data availability. Bold leadership, like that which launched the GPEI and continues to guide it, cannot be paralyzed by uncertainty. The hope is that the threats examined by McCarthy et al. [3], as well as those related to silent circulation at the time that OPV is stopped, never materialize. Nevertheless, the huge costs of failure justify greater efforts to make GPEI data available to the emerging cadre of scientists who can determine how to minimize risks.

## Conclusions

Modeling can uncover threats to polio eradication and present the most cost-effective paths to overcoming these threats. The two papers in *BMC Medicine* discussed herein make significant advances in this regard. However, their methods cannot fully eliminate the key uncertainties that could threaten eradication when OPV use is stopped. Newly developed methods are therefore needed. The complex nature of inference using these methods requires analyses by multiple groups with diverse perspectives and methodological skills. However, the needed analyses will not happen without direct accessibility to GPEI data.
